# High-content behavioral profiling reveals neuronal genetic network modulating *Drosophila* larval locomotor program

**DOI:** 10.1186/s12863-017-0513-7

**Published:** 2017-05-12

**Authors:** Boanerges Aleman-Meza, Mario Loeza-Cabrera, Omar Peña-Ramos, Michael Stern, Weiwei Zhong

**Affiliations:** Department of BioSciences, Rice University, Houston, TX 77005 USA

**Keywords:** High-content phenotypic profiling, Genetic interaction network, Larval locomotion, Behaviour, *Drosophila melanogaster*

## Abstract

**Background:**

Two key questions in understanding the genetic control of behaviors are: what genes are involved and how these genes interact. To answer these questions at a systems level, we conducted high-content profiling of *Drosophila* larval locomotor behaviors for over 100 genotypes.

**Results:**

We studied 69 genes whose *C. elegans* orthologs were neuronal signalling genes with significant locomotor phenotypes, and conducted RNAi with ubiquitous, pan-neuronal, or motor-neuronal Gal4 drivers. Inactivation of 42 genes, including the nicotinic acetylcholine receptors *nAChRα1* and *nAChRα3*, in the neurons caused significant movement defects. Bioinformatic analysis suggested 81 interactions among these genes based on phenotypic pattern similarities. Comparing the worm and fly data sets, we found that these genes were highly conserved in having neuronal expressions and locomotor phenotypes. However, the genetic interactions were not conserved for ubiquitous profiles, and may be mildly conserved for the neuronal profiles. Unexpectedly, our data also revealed a possible motor-neuronal control of body size, because inactivation of *Rdl* and *Gαo* in the motor neurons reduced the larval body size. Overall, these data established a framework for further exploring the genetic control of *Drosophila* larval locomotion.

**Conclusions:**

High content, quantitative phenotyping of larval locomotor behaviours provides a framework for system-level understanding of the gene networks underlying such behaviours.

**Electronic supplementary material:**

The online version of this article (doi:10.1186/s12863-017-0513-7) contains supplementary material, which is available to authorized users.

## Background

A key challenge in neurobiology is to connect behaviors to neurons to genes. The fruitfly *Drosophila melanogaster* larva is a powerful model for research in this area. Quantitative studies of the larval locomotor behaviors, such as [1–6], have enabled many discoveries linking genes, neurons, and phenotypes. Recent technologies in high-throughput phenotyping have advanced research in this area from a single gene level to a systems level. For example, a multi-larvae tracking system [7], in combination with optogenetics and a vast collection of Gal4 drivers, have enabled discovery of the neuronal circuits regulating behaviors such as peristaltic crawling [8] and decision-making in response to a mechano-stimulus [9].

Connecting behaviors to gene networks remains largely unexplored at the systems level even for this relatively simple animal. To understand the gene networks, one must reveal which genes are involved (nodes), and how they interact (edges). The major challenge is how to map genetic interactions. One method to reveal genetic interactions is epistasis analysis [10], which compares double and single mutant phenotypes to identify enhancing and suppressing effects. In *Drosophila*, this method requires genetic crosses to generate double mutants, and is thus not practical for large-scale mapping of many genes. In such cases, high-content profiling becomes an effective method in mapping genetic interactions. Genes with similar phenotypic profiles are considered interacting. In animals, high-content profiling was successfully applied to discover gene interaction networks regulating *C. elegans* embryogenesis [11], gonad architecture [12], and locomotor behaviors [13, 14].

We have recently developed the imaging system MaggotTracker to analyse multiple parameters of *Drosophila* larval locomotor behaviors [15]. MaggotTracker tracks a single animal at a high resolution and measures over 20 parameters, enabling high-content profiling of *Drosophila* phenotypes.

Here we present high-content larval locomotor profiles for 69 genes using the MaggotTracker. These profiles revealed 42 genes whose inactivation in the neurons caused significant movement defects, and 81 genetic interactions among these genes. As we focused on *Drosophila* genes whose *C. elegans* orthologs are locomotor genes, this data set also revealed that while many genes are conserved in their involvement in locomotor behaviors and neuronal expression, the genetic interactions were conserved to a much lesser degree. Finally, our data suggested a motor-neuronal control of body length, as inactivation of *Rdl* and *Gαo* in motor neurons caused reduced larval size.

## Methods

### Animals

Fly strains were obtained from the Bloomington *Drosophila* Stock Center (IN). All stocks were maintained on cornmeal agar following the Bloomington food recipe (0.8% yeast, 0.93% soy flour, 6.79% yellow cornmeal, 0.8% agar, 7.1% Karo light corn syrup, and 0.45% propionic acid). Animals were cultured at room temperature (~22 °C) for regular stock maintenance. All RNAi strains are listed in Additional file 1: Table S1. The Gal4 strains used in this study were *D42-Gal4* [16], *elav-Gal4* [17], *dilp2-Gal4* [18], *tub-Gal4* (Bloomington #5138), and *da-Gal4* (#108252) which was provided by the *Drosophila* Genetic Resource Center.

### Genetic crosses

Males from the UAS-dsRNA strain and virgin females from the Gal4 driver strain were crossed. A strain expressing UAS-dsRNA for mCherry RNAi (stock # 35785) was used as control. For RNAi strains in which the CyO balancer was present in the stock, the CyO was replaced with CyORoi, and males with smooth eyes (lacking the balancer) were used in the cross. The stock number and RNAi gene for these balanced strains are #38966, *CanA-14F*; #44014, *Syb*; #44538, *CG1909*; #44539, *robo3*; #51049, *nAChRα7*; #53296, *unc-104*.

Two RNAi stocks (#28574, *RSG7*; #36746, *CG31140*) carried the TM3 balancer. In crosses involving these two strains, larvae were recovered after tracking, placed in a 24 well plate containing cornmeal agar (1 larva per well) and left at 20 °C until they became adult flies. Then each fly was inspected to determine if it carried the balancer. Videos of the larvae that later showed the stubble phenotype (carrying the TM3 balancer) were discarded.

The default culture temperature for genetic crosses was 25 °C. That is, while parental strains were maintained at room temperature (~22 °C), genetic crosses, which included placing males and females from two different parental strains together, and subsequent larval growth, were kept at 25 °C. If RNAi caused lethality, crosses were kept at 20 °C. For crosses using the *tub-Gal4* driver, if 20 °C still caused lethality, then *da-Gal4* driver was used. Similarly, *da-Gal4* driver was tested at 25 °C first and reduced to 20 °C if there was lethality. At least 3 independent crosses were conducted for each strain. Animals from these crosses were pooled prior to analysis.

### Behavioral assay

Animals were tracked using the MaggotTracker as described [15]. Briefly, animals were placed at 20 °C for at least 12 h before third instar wandering larvae were collected using a paintbrush. Animals were examined under a dissecting scope to determine the gender and confirm the absence of food. A larva was then placed on a 10 cm Petri dish plate containing 1.5% agar, and left on the plate for at least 30 s so that they could acclimate to the media. The animal was then tracked for 4 min using the computer-controlled system. At least 10 males and 10 females were tracked for each genotype. At least 10 control animals (5 males and 5 females) were tracked in every tracking session.

### Statistical analysis

Mutant parameter values were normalized by the mean values of control animals tested in the same experiment. Mutant locomotive profiles were compared with control ones using the Student’s t-test. Benjamini-Hochberg procedure [19] was applied to correct multiple comparisons to control the false discovery rate (FDR) below 1%.

### Clustering and network visualization

Gene Cluster 3.0 [20] was used to perform hierarchical clustering with centroid linkage. Java TreeView [21] was used to display the clusters. Network visualization was performed using the software GUESS (graphexploration.cond.org).

### A strategy for high-content phenotyping of larval locomotor behaviors

**Fig. 1:**
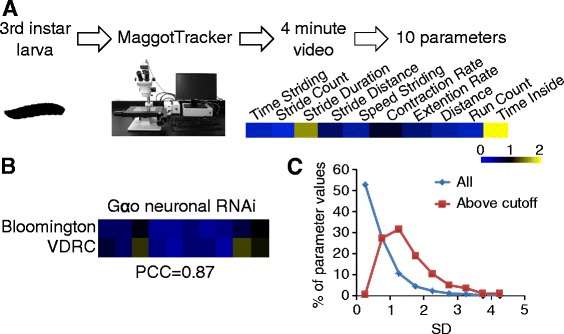
A strategy to conduct high-content profiling of larval locomotor behaviors. **a** Third instar larvae of different genotypes were analyzed using the MaggotTracker imaging system. Each larva was tracked and recorded for 4 min. Parameters were extracted from the videos. Mutant values were divided by control values to obtain normalized values. A heatmap was used to display normalized mutant parameter values, with *blue*, *black*, *yellow* indicating mutant values <, =, > 1, respectively. The same color scale was used for all figures. **b** *Gαo* neuronal RNAi produced similar phenotypes using UAS-RNAi strains from two stock centers. PCC, Pearson Correlation Coefficient. **c** Histograms of Z scores from parameter values of all genotypes (All) and parameter values that were considered significantly different than wild-type values (Above cutoff). SD, Standard deviation. Z scores measure how much a mutant value deviate from the wild-type value in SDs

A research pipeline was designed to conduct high-content profiling of larval locomotive behaviors for various mutants (Fig. 1a). We have developed an imaging system, MaggotTracker [15], to automatically track a single fruit fly larva, take a video, and extract 20+ parameters from the video measuring different aspects of larval crawling. Each third instar larva was tracked for 4 min. 10+ males and 10+ females were tracked for each genotype so that possible sexual dimorphism would be detected.

The phenotypic profiles contained 10 parameters (Table 1) that were selected because they were independent and had similarly low variance [15]. As the parameters were in different units, a normalization process was conducted. Values from mutants were divided by mean values from wild-type animals tracked on the same date to obtain normalized values. This normalization step also reduced day-to-day environmental variations [15]. In the normalized profiles, 1 indicates wild-type value for all parameters; <1 and >1 indicate lower and higher than wild-type value, respectively.

**Table 1:** Parameters of the locomotor profiles

Parameter	Unit	Definition of Parameter
Time Striding	%	Percentage of time when the larva is striding (continuous peristaltic movement).
Stride Count	counts/min	Total number of strides.
Stride Duration	sec	Time duration of one stride.
Stride Distance	mm	Distance traveled by the center point of the animal during one stride.
Speed Striding	mm/s	Speed of the center point of the body when the animal is striding.
Contraction Rate	mm/s	The rate of body length change during the contraction phase of a stride.
Extension Rate	mm/s	The rate of body length change during the extension phase of a stride.
Distance	mm/min	Total distance traveled by the center point.
Run Count	counts/min	Total number of runs. A run is defined as a period when the animal is striding continuously.
Time Inside	%	Percentage of time the animal is inside the plastic ring that was placed on the outer rim of the agar plate to prevent the animal from crawling to the edge.

## Results

### High-content behavioral profiles of neuronal signalling genes

To select candidate genes for testing, we took advantage of a previous high-content phenotyping study of *C. elegans* locomotor behaviors. We have previously screened 227 worm neuronal signalling genes that had homozygous-viable mutants, and found 87 genes with significant locomotor defects [14]. We mapped the *D. melanogaster* orthologs of these 87 genes. Among the orthologs, 69 genes have RNAi strains available at the Bloomington stock center (Additional file 1: Table S1). These genes became our candidate genes.

We crossed the UAS-RNAi strains [22] with several GAL4 driver strains to inactivate the gene function in different tissues. A ubiquitous *tub-Gal4* driver was used to detect gene function in the entire larvae. If the RNAi resulted in larval lethality, then a weaker ubiquitous driver *da-GAL4* was used instead. All genes were also inactivated in the neurons using a pan-neuronal *elav-GAL4* driver. Selected genes were inactivated using additional GAL4 drivers. For example, for genes that showed strong locomotor phenotypes upon neuronal inactivation, a motor-neuron specific *D42-GAL4* driver was used to examine whether these genes function in the motor neurons.

Using the imaging system MaggotTracker (Fig. 1a, also see Method), we obtained high-content locomotive profiles for 128 genotypes of pan-neuronal or ubiquitous RNAi after analysing over 3600 videos of individual animals (Additional file 2: Table S2). Among the 69 genes we tested, excluding those whose RNAi caused larval lethality, neuronal RNAi profiles were obtained for 68 genes, and ubiquitous RNAi profiles were obtained for 60 genes. Upon neuronal RNAi, 42 genes showed significant larval locomotor defect in at least one parameter (FDR < 0.05); upon ubiquitous RNAi, 37 genes showed significant locomotor phenotypes (Additional file 2: Table S2). Combined, significant larval locomotor defects were observed for 58 genes in either ubiquitous or neuronal RNAi; 21 genes showed defects in both types of RNAi. It should be noted that lack of a phenotype could be a consequence of inadequate knockdown.

Several data suggested the validity of this RNAi screen. We randomly selected one gene, Gαo, and tested its neuronal RNAi using a UAS-RNAi strain from the VDRC stock center. This strain showed a phenotype highly similar to that of the UAS-RNAi strain from the Bloomington stock center (Fig. 1b), suggesting that the phenotype is gene specific. In addition, among the genes with RNAi larval locomotor phenotypes, mutants of *cac*, *CanA1*, *Caps*, *dys*, and *norpA* are known to have locomotor defects in adults (FlyBase). The consistency between our RNAi results with known locomotor effects of chromosomal mutations suggested that the RNAi effects are likely gene specific.

The sensitivity of our phenotyping is high because of the quantitative measurements. Among the parameter values that we found to be significantly different than wild-type values, most of them were 0.5–2 standard deviations (SDs) away from wild-type values (Fig. 1c). 91% of these 58 genes identified with larval locomotor defects in this study have not been previously associated with any locomotor defects. Among the 68 genes for which we had locomotor profiles, only 6 genes were annotated with locomotor defects with any mutant allele at any developmental stage in FlyBase (flybase.org, version FB2016_05). Our phenotyping data recaptured 5 of the 6 genes to have significant larval locomotor defects, and detected 53 more genes with such phenotype. Together, these data suggested that our current knowledge on genetic control of larval locomotion is still largely incomplete, and that our method is highly effective in discovering genes with such functions.

### Neuronal genes required for larval locomotion

To understand how genes function in the neurons to regulate larval locomotion, we seek answers to two questions: what genes are involved, and how these genes interact.

**Fig. 2:**
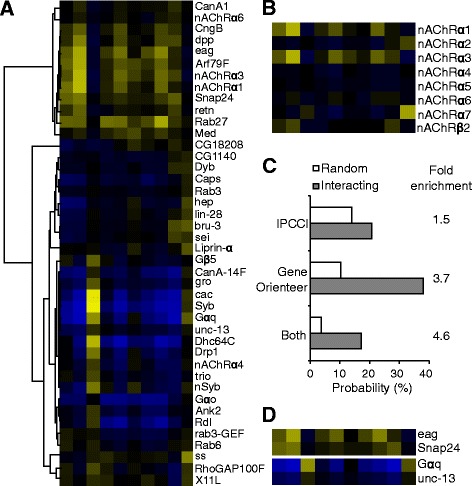
Genes required in the neurons to regulate locomotor behaviors. **a** Hierarchical clustering of the 41 neuronal profiles with significant phenotypes. **b** Neuronal profiles of nAChRs. **c** Known interacting genes are enriched with correlated profiles (|PCC| > 0.7) and high GeneOrienteer scores. **d** Neuronal profiles of the four genes that have high |PCC| and GeneOrienteer scores with *nAChRα1* and *nAChRα3*

RNAi of the following 42 genes in the neurons caused significant larval locomotor defects: *Ank2*, *Arf79F*, *bru-3*, *cac*, *CanA-14F*, *CanA1*, *Caps*, *CG18208*, *CG31140*, *CngB*, *Dhc64C*, *dpp*, *Drp1*, *Dyb*, *eag*, *Gαo*, *Gαq*, *Gβ5*, *gro*, *hep*, *lin-28*, *Liprin-α*, *Med*, *nAChRα1*, *nAChRα3*, *nAChRα4*, *nAChRα6*, *nSyb*, *Rab27*, *Rab3*, *Rab3-GEF*, *Rab6*, *Rdl*, *retn*, *RhoGAP100F*, *sei*, *Snap24*, *ss*, *Syb*, *trio*, *unc-13*, *X11L* (Fig. 2a, Additional file 2: Table S2). Among them, an interesting case is the nicotinic acetylcholine receptors (nAChRs). Mutations in human nAChRs are linked to a range of diseases such as epilepsy and autoimmune diseases [23]. There are 10 nAChRs in the *Drosophila* genome, α1-α7 and β1-β3 [24]. These nAChRs are expressed in the nervous system and are targets of insecticides [24]. Their in vivo physiological functions are largely unknown except that α7 is required for an escape behavior [25].

Eight nAChRs, α1-α7 and β2, have RNAi strains available at the Bloomington center and were tested in this study. Neuronal inactivation of α1 and α3 produced most severe and very similar larval locomotor phenotypes (Fig. 2b). It was reported that mutants of *nAChRα1* and *nAChRβ2* showed resistance to the neonicotinoid class of insecticides [26]. Consistent with this, neuronal RNAi of *nAChRβ2* had a larval locomotor profile highly similar to that of *nAChRα1*, although the phenotype of *nAChRβ2* is weaker than that of *nAChRα1* (Fig. 2b). These data indicated that among the nAChRs, α1, α3 and β2 share similar functions in regulating larval locomotive behaviors. It was shown that *nAChRα5* and *nAChRα7* can form heteromeric ion channels [27]. It is possible that α1, α3 and β2 may also form heteromeric ion channels.

### Interactions of neuronal locomotor genes

To identify the interactions among the 42 neuronal larval locomotive genes, we computed the Pearson correlation coefficient (PCC) to evaluate the similarity of phenotypic patterns. We reasoned that interacting gene pairs were more likely to have similar (PCC close to 1) or opposite (PCC close to −1) phenotypic patterns, resulting a high absolute value of PCC (|PCC| close to 1). In contrast, if two phenotypic patterns were not correlated at all (|PCC| close to 0), then less likely these two genes would interact. To verify such rationale, we queried the databases BioGrid (thebiogrid.org) and FlyBase for known interactions. Among all genes we tested, there were 29 gene pairs known to interact genetically or physically. Indeed, these interacting genes were more likely to have high |PCC| values of their locomotive profiles. 21% of interacting gene pairs had |PCC| value over 0.7 for their locomotive profiles, whereas only 14% of random parings of neuronal profiles did (Fig. 2c), confirming that |PCC| could be used to distinguish interacting genes. The 42 neuronal larval locomotive genes generated 861 (42 × 41/2 = 861) pairwise combinations. Among them, a total of 302 pairs showed |PCC| above 0.7.

**Fig. 3:**
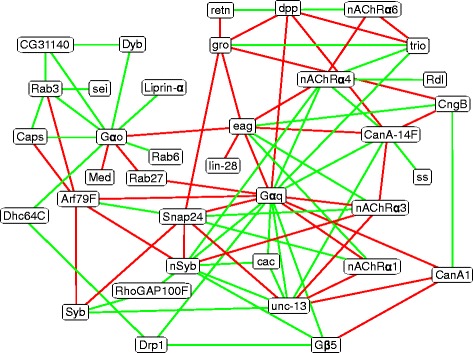
High-confidence neuronal gene interactions. *Green* edges indicate two genes have similar behavioral profiles; *red* edges indicate two genes have opposite behavioral profiles

To further prioritize these 302 gene pairs for genetic interactions, we queried GeneOrienteer (geneorienteer.org), a database that predicts genetic interactions by integrating expression, phenotype, gene ontology, and interaction data from multiple species [28]. Our previous investigation of *C. elegans* locomotive genes showed genetic interactions were most enriched among gene pair that satisfied two conditions: |PCC| above 0.7, and GeneOrienteer score over 4 [14]. A similar result was observed in this study of fly genes: interacting pairs were 4.6 times more likely than random pairing of locomotor profiles to satisfy both criteria, while individual criteria could only provide 1.5 and 3.7 times enrichment (Fig. 2c). Two hundred twenty-two gene pairs among the 42 neuronal larval locomotive genes had GeneOrienteer scores over 4. Using both |PCC| and GeneOrienteer scores as criteria, 81 pairs of neuronal larval locomotive genes were identified as high-confidence interactions (Additional file 3: Table S3, Fig. 3).

Some of these 81 high-confidence interactions are consistent with known interaction data. For example, the gene products of two pairs, *unc-13* – *Syb* and *Snap24* – *Syb*, have been shown to interact physically in yeast two-hybrid experiments (BioGrid). The yeast orthologous pair of *Arf79F* – *Syb* and the human orthologous pair of *Snap24* – *unc-13* also encode proteins that physically interact (BioGrid). The orthologs of 13 pairs interact genetically in *C. elegans*, including *unc-13* – *Syb, Gαq* – *Gbeta5, CG31140* – *Gαo, nSyb* – *unc-13*, *Gαq* – *trio*, *Snap24* – *Syb*, *Gαq* – *unc-13*, *CanA-14F – Gαq*, *CanA1 – Gαq*, *Caps – Gαo*, *Gαo – Liprin-α*, *nSyb – RhoGAP100F*, *RhoGAP100F – Syb* (WormBase). These data demonstrated the validity of these high-confidence interactions.

The high-confidence interaction network shows that *nAChRα1* and *nAChRα3* interact with the SNARE protein *Snap24*, and the potassium channel *eag*, with similar phenotypic patterns; *nAChRα1* and *nAChRα3* also interact with the synaptic vesicle protein *unc-13*, and the G protein *Gαq*, with opposite phenotypic patterns (Figs. 2d, 3). It was reported that *unc-13* functions downstream of *Gαq* signalling, and upstream of vesicular fusion at the neuromuscular junction (NMJ) [29, 30]. Our data suggested that *eag* and nAChRs have an antagonistic effect on this *Gαq* signalling pathway in locomotor behaviors. The involvement of *Snap24* suggested that the antagonistic effect on *Gαq* signalling is possibly mediated through neurotransmitter release or reuptake.

### Site-of-action for larval locomotive genes

To understand whether the locomotor genes primarily function in neurons or in additional tissues, we compared the locomotive profiles from pan-neuronal RNAi with those from ubiquitous RNAi. If inactivating the gene in the neurons generates the same phenotype as inactivating the gene ubiquitously, then the gene function is most likely to be neuronal. As RNAi strength varies with different Gal4 drivers, the phenotypic severity of different RNAi may differ, causing difference in our parameter values. Therefore, we evaluated the PCC of profiles because phenotypic patterns are less affected by RNAi strength.

**Fig. 4:**
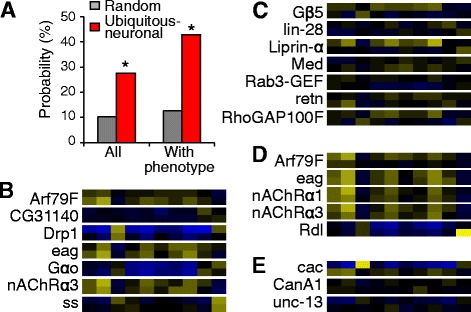
Locomotor genes site-of-action. **a** The ubiquitous and neuronal profiles from the same gene is more likely to have PCC > 0.7 than randomly paired profiles. In one data set all profiles were tested; in the other, only profiles with significant phenotypes were tested. *, *p* < 0.01, Fisher’s exact test. **b** Genes with correlated (PCC > 0.7) ubiquitous (top) and pan-neuronal (bottom) behavioral profiles. **c** Genes with uncorrelated (PCC < 0.1) ubiquitous (top) and pan-neuronal (bottom) behavioral profiles. **d** Genes with correlated pan-neuronal (top) and motor-neuronal (bottom) behavioral profiles. **e** Genes with uncorrelated pan-neuronal (top) and motor-neuronal (bottom) behavioral profiles

Pan-neuronal RNAi profiles were more likely to be correlated with ubiquitous RNAi profiles of the same genes. 26% genes had highly correlated (PCC > 0.7) ubiquitous and neuronal profiles; among genes with significant locomotor phenotypes in both neuronal and ubiquitous RNAi, 38% had PCC > 0.7 for ubiquitous and neuronal profiles (Fig. 4a). These data suggested that many genes we tested predominantly function in the neurons. For example, *Arf79F*, *CG31140*, *Drp1*, *eag*, *Gαo*, *nAChRα3*, *Rab 27*, and *ss*, had highly correlated ubiquitous and neuronal profiles (Fig. 4b), suggesting that these genes function primarily in the neurons to regulate locomotion. In contrast, *Gβ5*, *lin-28*, *Liprin-α*, *Med*, *rab3-GEF*, *retn*, *RhoGAP100F*, had little correlation (PCC < 0.1) between their ubiquitous and neuronal profiles (Fig. 4c), suggesting that these genes have functions in non-neuronal tissues as well as neurons to regulate larval locomotion.

For the genes with severe neuronal phenotypes, we investigated whether they primarily function in motor neurons. The behavioral profiles from the pan-neuronal *elav-Gal4* driver were compared with those from the motor-neuronal *D42-Gal4* driver. *Arf79F*, *eag*, *nAChRα1*, *nAChRα3*, and *Rdl* showed highly correlated (PCC > 0.7) pan-neuronal and motor-neuronal profiles (Fig. 4d), suggesting that these genes primarily function in the motor neurons to regulate larval locomotion. *Drp1*, *Gαo*, *Gαq* showed moderately correlated (PCC between 0.6–0.7) pan-neuronal and motor-neuronal profiles. These genes may still primarily function in the motor neurons. *Cac*, *CanA1*, and *unc-13* had uncorrelated (PCC < 0.3) pan-neuronal and motor-neuronal profiles (Fig. 4e), suggesting that these genes have functions in neurons other than motor neurons. The requirement of different neurons in larval locomotor regulation is consistent with previous findings such as [8, 31].

For the nAChRs, *nAChRα1* and *nAChRα3* showed highly similar phenotypic profiles with pan-neuronal, and motor-neuronal RNAi (Fig. 4d), suggesting that these genes primarily function in motor neurons. Similarly, their interacting genes, *Gαq* and *eag*, are also likely to primarily function in motor neurons (Fig. 4d). In contrast, the interactor *unc-13* is likely to function in other neurons and non-neuronal tissues (Fig. 4c, e).

### Conservation of gene functions in locomotor phenotypes and neuronal expression

Because all genes we tested in this study were orthologs of *C. elegans* genes that had been analyzed previously for their locomotor defects [14], such data enabled us to compare gene functions in *C. elegans* and *D. melanogaster* to understand their levels of conservation. Three aspects of gene functions were evaluated: expression, phenotype, and gene interactions. The two animals have different nervous system anatomy and different locomotive pattern: *C. elegans* employs a sinusoidal wave of crawling whereas fly larvae employ peristalsis crawling. Therefore, the detailed cellular gene expression pattern and locomotive parameters are not comparable between the two species. However, we can compare gene function at a higher level such as whether the genes are neuronally expressed and whether they affect movement.

**Fig. 5:**
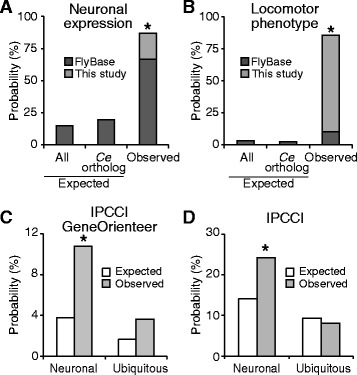
Conservation and rewiring of genetic control of locomotor behaviors between worms and flies. **a** The examined set was enriched with genes expressed in the neurons. **b** The examined genes were enriched with locomotor phenotypes. In (**a**) and (**b**), expected background values were computed using Flybase annotations for all genes (All) or genes with *C. elegans* orthologs (*Ce* ortholog). *, *p* < 0.01 comparing observed value (FlyBase alone, or combined data from FlyBase and this study) with both expected values, Fisher’s exact test. **c**, **d** Mild conservation of genetic interactions, if interaction was defined using both |PCC| for locomotive profiles and high GeneOrienteer scores (**c**) or only |PCC| values (**d**). Both neuronal and ubiquitous profiles were analyzed. Observed values were computed using fly gene pairs with interacting worm orthologous pairs. Expected values were computed using all combinations of genes. * indicates *p* < 0.01 comparing observed and expected values, Fisher’s exact test

All orthologous worm genes were annotated as neuronal genes in WormBase. We queried FlyBase and found that 67% (46/69) genes were also annotated as neuronally expressed, i.e., associated with terms such as brain, nerve, neuron, or nervous system. In contrast, only 15% of all genes in the fly genome and 19% of all fly genes with worm orthologs were annotated as expressed in the neurons (Fig. 5a). In addition to FlyBase annotations, 12 genes that were not annotated in FlyBase as neuronally expressed showed locomotor phenotypes in this study when inactivated in the neurons, suggesting that they function in the neurons. Combined, 87% (60/69) of our tested fly genes are neuronal genes (Fig. 5a), demonstrating a high level of conservation in gene expression.

In addition to expression, these genes also showed a high level of conservation in phenotypes. All orthologous worm genes had mutants with significant locomotor defects [14]. In this study, upon RNAi inactivation, 85% (59/69) of our tested fly genes showed locomotor phenotypes (Fig. 5b). We further queried FlyBase to estimate how likely a randomly selected gene would display a locomotor phenotype. Among FlyBase phenotype annotations, only 3.1% of all genes in the fly genome and 2.5% of all genes with worm orthologs were annotated with locomotor defects (Fig. 5b). This ratio significantly (*p* < 0.01, Fisher’s exact test) increased to 10% among our tested genes (Fig. 5b), demonstrating that locomotor defects are enriched among these genes. Together, these data suggested a strong conservation among these genes in locomotor phenotypes.

### Rewiring of genetic interactions among locomotor genes

Next we examined the conservation of genetic interactions. Worm interacting gene pairs were defined as genes with |PCC| > 0.7 for worm locomotive profiles and GeneOrienteer score over 4 [14]. For fly gene pairs whose worm orthologous pairs were interacting, 11% were interacting if we used the same criteria for fly gene interactions and if we used locomotive profiles from neuronal RNAi (Fig. 5c). While such conservation level was drastically lower than those in expression or phenotype (11 vs. ≥85%), it is significantly (*p* < 0.001, Fisher’s exact test) higher than the expected probability from random gene pairs (11 vs. 4%, Fig. 5c). In comparison with neuronal locomotive profiles, profiles from ubiquitous RNAi showed even a lower level of conservation: Fly gene pairs with interacting worm orthologous pairs had only 2.4% probability of interacting, which was not significantly higher than the 1.2% from random pairs (Fig. 5c).

We investigated whether our results on genetic interactions were biased by the GeneOrienteer algorithm as it used orthologous data. When we eliminated GeneOrienteer scores and used |PCC| values as the sole criterion for interaction, we observed that 24% of genetic interactions were conserved if neuronal profiles were used, which was significantly higher than the expected 14% from random pairs (Fig. 5d). Conservation of genetic interactions was not observed in ubiquitous profiles (Fig. 5d). These data confirmed that the conservation level of genetic interactions was much lower than those of expression and phenotype.

### Motor-neuronal control of body length

**Fig. 6:**
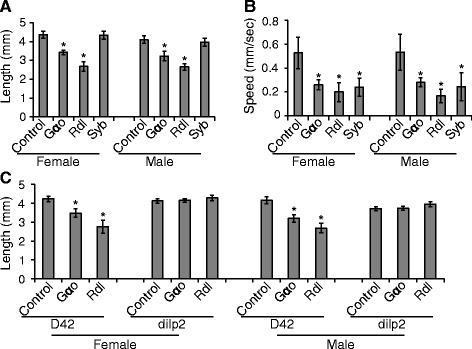
Motor neuronal control of body size. **a** RNAi of *Gαo* and *Rdl*, but not *Syb*, caused smaller body size in both males and females. **b** The locomotor defects are similarly severe for neuronal RNAi of *Gαo*, *Rdl*, and *Syb*. **c** RNAi of *Gαo* and *Rdl* in motor neurons (*D42-Gal4* driver), but not insulin-producing neurons (*dilp2-Gal4* driver) recaptured the smaller body size phenotype. *, *p* < 0.01, Student’s t-test. *n* ≥ 10 animals for each genotype. Bars and error bars indicate mean and standard deviation

While we used only 10 locomotive parameters in this study, our imaging system extracts 20+ parameters from the videos. One parameter that was measured by the system but not used in the locomotive profiles was body size. As male larvae are smaller than female larvae, we analyzed them separately in all body length measurements. Notably, inactivation of two genes, *Rdl* and *Gαo*, in neurons caused significantly reduced larval body size in both females and males (Fig. 6a).

As neuronal RNAi of *Rdl* and *Gαo* also caused severe locomotive defects, it seemed possible that the reduced body length might be a secondary defect of the locomotive phenotypes. For example, if a larva could not move well, it might not reach food well, and thus could have a small body length because of malnutrition. This was unlikely under our culture conditions as the larvae were maintained continuously on food. Furthermore, neuronal RNAi of many genes caused severe locomotor defects, without affecting body length. For example, neuronal RNAi of *syb* reduced the larval crawling speed to a level similar to *Rdl* and *Gαo* (Fig. 6b), yet caused no body length defects (Fig. 6a). Therefore, we hypothesize that the reduced larval length of *Rdl* and *Gαo* reflects a primary defect in gene function.

Surprisingly, inactivating *Rdl* and *Gαo* in motor neurons using the *D42-Gal4* driver reproduced the shortened-body-length phenotype (Fig. 6c). In contrast, inactivating these genes in insulin producing brain cells using a *dilp2-Gal4* driver did not cause reduced body length (Fig. 6c). These data suggested that there is a motor-neuronal control of body length in *D. melanogaster*, and that such control requires the function of *Rdl* and *Gαo* in motor neurons.

### Database

In addition to the supplementary tables, an interactive database was built to enable queries of videos and behavioral profiles including all 20+ parameters. The database provides raw measurements as well as normalized measurements of each animal for query and download. The database can be accessed at http://wormloco.org, the same website that also stores *C. elegans* locomotor behavioral profiles [14].

## Discussion

We present here the high-content larval locomotive profiles of 69 genes whose *C. elegans* orthologs are neuronal signalling genes with locomotor phenotypes. Profiles were collected after inactivation of these genes by RNAi in all cells, neurons, and motor neurons. Bioinformatic analysis on these profiles revealed 42 genes whose functions are required in the neurons to maintain normal larval locomotor behaviors, and 81 high-confident interactions among these genes. While many of these genes are known to be expressed in neurons and have adult behavioral phenotypes, the difference between our study and the previous findings are: 1) this study revealed the functions of these genes in larval instead of adult behaviors; 2) this study provided quantitative, high-content phenotypic measurements instead of qualitative observations or single-metric measurements.

Using the nAChR family as an example, we showed how such analysis brings insights to gene functions. Our profiles revealed that inactivation of *nAChRα1* and *nAChRα3* in the neurons caused severe movement disorders. The interaction analysis suggested that these two nAChRs function similarly with *Snap24* and *eag*, but antagonistically with *Gαq* and *unc-13*. Site-of-action analysis suggested that *nAChRα1*, *nAChRα3*, *eag*, *Gαq* function primarily in the motor neurons. These results are consistent with previous reports that *Gαq* and *unc-13* function in the same signalling pathway in NMJ [29, 30]. It was reported that cholinergic input directly stimulates motor neurons [32], consistent with our finding that nAChRs are required in the motor neurons to maintain normal movement.

While high-content profiling cannot definitively prove genetic interactions as epistasis analysis, the effectiveness of this method has been well established [11–14]. As there is no high-throughput method to epistasis analysis in flies, experimental validation of these predicted genetic interactions is difficult. We thus relied on a computational validation of the predicted genetic interactions, and showed that the predicted interactions are enriched among the known positives (known interactions) than negatives (random gene pairs). Random gene pairs were used as negatives because there is no sizable data set on genes that do not interact, and because genetic interactions are sparse among all gene pairs.

It has remained unknown whether genetic interaction networks are conserved for multicellular organisms. This is mostly due to lack of large-scale genetic interaction studies: the only available multicellular in vivo genetic interaction networks are synthetic lethality networks in *C. elegans* [33, 34]. While certain conservation was observed for genetic interaction networks between budding and fission yeast, no conservation was observed between these worm networks and the yeast ones [35, 36]. Using the same methodology of high-content profiling, we provided comparable data sets to estimate the conservation of genetic interaction networks in worms and flies. Similar to the worm-yeast result, no strong conservation of genetic interactions between worms and flies was observed under ubiquitous RNAi. *C. elegans* and *Drosophila* larvae have different nervous system and muscle anatomy. As genetic interactions can involve genes functioning in different cells, the difference in cellular circuitry may explain the lack of conservation. Similar reasoning may also explain the mild conservation between worm and fly neuronal genetic interactions (11% conservation rate, Fig. 4d), as these interactions are limited to the same tissue. Consistent with this hypothesis, this mild conservation rate is similar to the genetic interaction conservation rate observed between budding yeast *S. cerevisiae* and fission yeast *S. pombe*: 17% negative interactions and 10% positive interactions were conserved [36].

The observed motor neuronal control of body size was unexpected. One possible mechanism for such motor-neuronal effect of body size is through muscle cells. It was discovered that inhibition of insulin receptor (InR) in muscle cells could cause systemic effects and reduce the body size of the entire larvae [37] an effect that might be mediated at least in part by the Foxo-dependent increase in release of *ImpL2*, an insulin signalling inhibitor [38]. It is possible that *Rdl* and *Gαo* functions in motor neurons affect the muscle state, which subsequently changes the activities of these regulators in muscle cells, and impacts body size. Given that *Rdl* encodes the *Drosophila* GABA_A_ receptor, which mediates inhibitory synaptic transmission, the *Rdl* knockdown is expected to increase motor neuron activity and hence synaptic input onto the muscle; this increased synaptic input might activate muscle Foxo. Consistent with this model, it was reported previously that effects of altered synaptic transmission in *Drosophila* larvae on activity of muscle insulin signalling components [39].

It was shown that a coordinated action from both excitatory neurons, which release acetylcholine, and inhibitory neurons, which release GABA, is required to generate the sequential firing of motor neurons for the peristaltic movement [40]. The acetylcholine receptor (nAChR) and the GABA_A_ receptor (Rdl) are likely to function in different dendrites of motor neurons to mediate the excitatory and inhibitor synaptic transmission, respectively, to drive the rhythmic firing of motor neurons responsible for peristaltic movements.

We do not know whether motor neuronal control of body size also exists in *C. elegans*. Neuronal control of body size exists in *C. elegans*, as it was reported that gene functions in sensory neurons affect body size in *C. elegans* [41]. Mutants of the *C. elegans* orthologs of *Rdl* and *Gαo*, *unc-49* and *goa-1*, had a smaller body length (wormloco.org). However, the site-of-action for these genes in size-regulation is unknown in *C. elegans*.

## Conclusion

Our high-content profile data provided a framework for understanding the genetic control of larval locomotion. In addition to providing clues for individual genes functions, such system-level approach also enabled evaluation of conservation and rewiring of genetic interaction networks.

## Additional files

Additional file 1: Table S1. Genes studied. List of the gene names, FlyBase IDs of the genes, the Bloomington stock numbers of fly strains used, and the worm orthologs of the fly genes (XLSX 12 kb).

Additional file 2: Table S2. Locomotive profiles of genes with significant phenotypes. Profiles are composed of normalized values for ten locomotive parameters. Profiles for both neuronal and ubiquitous RNAi are listed. The summary page lists genes with significant phenotypes (XLSX 26 kb).

Additional file 3: Table S3. Genetic interactions inferred from locomotive profiles. Predicted genetic interactions with |PCC| > 0.7 and GeneOrienteer score over 4 (XLSX 12 kb).

## Abbreviations

FDR: False discovery rate
InR: Insulin receptor
nAChR: nicotinic acetylcholine receptor
NMJ: Neuromuscular junction
PCC: Pearson correlation coefficient
SD: Standard deviation
